# Social Franchising and a Nationwide Mass Media Campaign Increased the Prevalence of Adequate Complementary Feeding in Vietnam: A Cluster-Randomized Program Evaluation^1^,^2^,^3^

**DOI:** 10.3945/jn.116.243907

**Published:** 2017-02-08

**Authors:** Rahul Rawat, Phuong Hong Nguyen, Lan Mai Tran, Nemat Hajeebhoy, Huan Van Nguyen, Jean Baker, Edward A Frongillo, Marie T Ruel, Purnima Menon

**Affiliations:** 4 Poverty, Health, and Nutrition Division, International Food Policy Research Institute, Washington, DC;; 5 Alive & Thrive, FHI 360, Washington, DC;; 6 Save the Children, Hanoi, Vietnam; and; 7 University of South Carolina, Columbia, SC

**Keywords:** complementary feeding, interpersonal counseling, mass media, child undernutrition, cluster-randomized trial, effectiveness evaluation, Vietnam

## Abstract

**Background:** Rigorous evaluations of health system–based interventions in large-scale programs to improve complementary feeding (CF) practices are limited. Alive & Thrive applied principles of social franchising within the government health system in Vietnam to improve the quality of interpersonal counseling (IPC) for infant and young child feeding combined with a national mass media (MM) campaign and community mobilization (CM).

**Objective:** We evaluated the impact of enhanced IPC + MM + CM (intensive) compared with standard IPC + less-intensive MM and CM (nonintensive) on CF practices and anthropometric indicators.

**Methods:** A cluster-randomized, nonblinded evaluation design with cross-sectional surveys (*n* = ∼500 children aged 6–23.9 mo and ∼1000 children aged 24–59.9 mo/group) implemented at baseline (2010) and endline (2014) was used. Difference-in-difference estimates (DDEs) of impact were calculated for intent-to-treat (ITT) analyses and modified per-protocol analyses (MPAs; mothers who attended the social franchising at least once: 62%).

**Results:** Groups were similar at baseline. In ITT analyses, there were no significant differences between groups in changes in CF practices over time. In the MPAs, greater improvements in the intensive than in the nonintensive group were seen for minimum dietary diversity [DDE: 6.4 percentage points (pps); *P* < 0.05] and minimum acceptable diet (8.0 pps; *P* < 0.05). Significant stunting declines occurred in both intensive (7.1 pps) and nonintensive (5.4 pps) groups among children aged 24–59.9 mo, with no differential decline.

**Conclusions:** When combined with MM and CM, an at-scale social franchising approach to improve IPC, delivered through the existing health care system, significantly improved CF practices, but not child growth, among mothers who used counseling services at least once. A greater impact may be achieved with strategies designed to increase service utilization. This trial was registered at clinicaltrials.gov as NCT01676623.

## Introduction

Appropriate infant and young child feeding (IYCF)^8^ practices, which include exclusive breastfeeding until 6 mo of age and the provision of safe and nutritionally rich foods in sufficient quantity, in addition to breast milk, from 6 to 23 mo of age, are a critical component of optimal child growth and development (1–3). Breastfeeding and complementary feeding (CF) promotion is recommended for scale-up in countries with a high burden of undernutrition (1). In Vietnam, an estimated 25% of children <5 y of age were stunted in 2014, reflecting a 5.8-percentage point (pp) decline since 2004. Over the same time period, there was little change in the prevalence of wasting, with an estimated prevalence of 6.8% in 2014, which reflects a decline of 1.1 pp in the previous 10 y (4). Poor IYCF practices is one of several factors explaining child undernutrition in Vietnam. Although breastfeeding is universal, practiced by 98% of mothers, only one-quarter of children aged 0–6 mo are exclusively breastfed (5). For CF practices, 2 key challenges identified in Vietnam are early introduction and low nutrient quality of CFs (6). In Vietnam, as elsewhere, strategies to improve IYCF are central to improving child nutrition.

Studies in a variety of contexts have reviewed strategies to improve child growth and development through investments in improving breastfeeding practices and CF knowledge and practices (2, 3, 7, 8). Small-scale efficacy trials to improve CF, including the provision of nutrition education and/or complementary foods, have shown positive impacts on maternal IYCF knowledge and child feeding practices (2), but the impacts on child growth are inconsistent (7, 9–12). Evidence of interventions to improve IYCF in Vietnam is scant and comes primarily from an integrated nutrition program that used the positive-deviance approach. This study showed positive impacts on the quantity of foods consumed and improvement toward meeting daily energy requirements of children but showed no impacts on child nutritional status (13).

At the time of planning this study, evidence of interventions to improve CF practices in low- and middle-income countries was derived predominantly from efficacy studies or small-scale or pilot effectiveness interventions. Programmatic examples of CF interventions delivered within the context of large-scale programs, or national health systems, with rigorous evaluation designs were limited.

As part of the Alive & Thrive (A&T) initiative, our multicountry portfolio included impact evaluations of 3 large-scale programs to examine the impacts of diverse models of improving IYCF, including breastfeeding and CF, in 3 countries: Bangladesh, Ethiopia, and Vietnam (14). In Bangladesh, the intervention consisted of outreach-based home counseling delivered by a nongovernmental organization (15). In Ethiopia, the CF counseling was delivered by outreach-focused governmental health system workers (16). In Vietnam, a health facility–based counseling approach was used. Mass media (MM) and community mobilization (CM) were used in all 3 countries to complement interpersonal counseling (IPC). Thus far, these studies have shown varying, but positive, impacts on breastfeeding in all 3 contexts (16, 17) and on CF in Bangladesh and Ethiopia (15, 16).

In this article, we report findings from Vietnam, where an innovative social franchise model (18) embedded into government health facilities was used to deliver interventions to improve CF and child growth. Social franchising applies commercial franchising concepts so that a brand identity is equated with quality services (in this case, through standardized operating procedures, support, and training) that help achieve social and health benefits (19). Studies on the ability to deliver and ensure quality of services for nutrition in health facilities are few (20, 21). These studies showed an impact on practices and on child growth outcomes, but scale-up of these interventions has been a challenge (22–24). This study therefore addresses an important gap in the literature on the impact of facility-based counseling interventions to address critical IYCF practices, particularly through the use of a social franchising–based model. The literature on social franchising itself, largely in the reproductive health field, is vast, but we are aware of no studies reporting experience with social franchising in the area of nutrition. This study therefore is the first to our knowledge to examine the role of social franchising to address IYCF practices and offers an example of the potential of the approach to address nutrition-related issues.

## Methods

### 

#### Study context and intervention description.

In Vietnam, A&T, through Save the Children, worked with the government to establish a total of 781 social franchises within government health facilities in 15 of 63 provinces at the province, district, and commune levels to improve the quality of IYCF counseling. Social franchising principles were used to deliver facility-based individual and group IYCF counseling, under the brand name Mặt Trời Bé Thơ (MTBT; “The Little Sun” in English). All facilities were required to meet minimum criteria, including a dedicated, standardized counseling room, trained staff, and availability of job aids and client materials. The program aimed to deliver a minimum of 9 and a maximum of 15 counseling contacts to each mother-child pair from the last trimester of pregnancy through the child’s first 2 y of life, among them 7 CF contacts from age 6 to 24 mo (**Table 1**). Referrals, CM, promotional print materials, and television advertising were used to generate demand for preventive IYCF counseling services, a concept that was new to most families. Training and supervision, incentives to the health facilities, and monitoring tools were applied to improve quality of services.

**TABLE 1:** Interpersonal counseling package^1^

	EBF promotion	EBF support	EBF management	CF education	CF management
Timing of contacts	Third trimester of pregnancy; 3 contacts: seventh month of pregnancy, eighth month of pregnancy, ninth month of pregnancy	Delivery; 1 contact	Child aged 0–6 mo; 4 contacts: at ages 2–4 wk, 1–2 mo, 2–3 mo, 4–5 mo	Child aged 5–6 mo; 1 contact	Child aged 6–24 mo; 6 contacts: at ages 6–7 mo, 8–9 mo, 10–11 mo, 12–14 mo, 15–18 mo, 18–24 mo
Types of counseling	2 individuals, 1 group	Individual	2 individuals, 2 groups	Individual	Combination of individual and group
Contents	Provides timely and appropriate information on EBF for mothers before delivery and in the third trimester of pregnancy	Supports mothers to initiate BF after delivery at health facilities	Follows up and supports mothers to maintain EBF in the 1–2 wk postpartum to 3–6 mo	Provides basic information needed for mothers to give appropriate complementary foods at 6 mo of age—not earlier or later	Provides information for mothers about CF, so mothers will: know age-appropriate CF practices, have the skills to practice age-appropriate CF, be able to prepare age-appropriate complementary foods, know appropriate foods to feed their infants by age, receive individual counselling that offers follow-up and support in CF
Principles of optimal CF	—	—	—	Start giving complementary foods at 6 mo of age, not too early or too late. Make foods tender for easy chewing and swallowing. Continue BF for as long as possible.	
				Start with liquids, then go to solid foods, from little to big amounts, to help the infant get acquainted with new food (not providing diluted food for >2 wk).	
				Increase the number of meals according to the child’s age; ensure that the food suits the infant’s appetite.	
				Prepare mixed foods rich in nutrients by using locally available foods.	
				Thicken the complementary food. Add oil, fat, sesame, or peanut in the complementary food to provide flavor and more energy and to help the infant grow fast.	
				Preparation and cooking tools must be clean; wash your hands before preparing meals and feeding the child.	
				Give the child more complementary food during and after the child’s illness and give the child more liquid food and drink, especially if the child has diarrhea or a high temperature.	
				Do not give the child MSG because it is not nutritious. Do not give the child confectionery or soft drinks before meals because the sweets increase blood sugar, inhibiting an extracting enzyme so that the child loses his or her appetite, skips the meal, or takes less food.	

1 BF, breastfeeding; CF, complementary feeding; EBF, exclusive breastfeeding; MSG, monosodium glutamate.

The MM component consisted of a national broadcast campaign that used television and digital media (Internet and mobile phone applications); 1 of 4 television spots focused on promoting iron-rich food consumption beginning at 6 mo of age and another television spot promoted the use of franchise services. In intensive areas, the MM campaign also included additional out-of-home advertising on optimal IYCF practices through billboards and broadcasts on village loudspeakers. In intensive areas, CM was operated by village health workers who visited households of women with children aged <24 mo to deliver invitation cards, encourage mothers to attend MTBT counseling services, and provide women with basic IYCF messages.

Thus, A&T used 3 different components (i.e., enhanced IPC, CM, and MM) to deliver interventions to targeted beneficiaries. The “intensive” group was exposed to all 3 interventions; the “nonintensive” group was exposed to standard IPC and less-intensive MM and CM. Standard IPC consisted of messages and information on IYCF delivered by doctors or midwives as part of routine child health care contacts at the health facilities. In nonintensive areas, the MM component did not include community airing of loudspeaker announcements and posters promoting breastfeeding in commune health centers. In nonintensive areas, CM was less structured and covered general health care topics, such as family planning, pregnancy registration, and antenatal care, and did not include the distribution of invitation cards to attend IYCF-related counseling. A more detailed description of the components of the intensive and nonintensive programs is provided elsewhere (18).

In Vietnam, IPC and MM were initiated in May and September 2011, respectively. Thus, the total duration of implementation of the full intensive package of interventions was ∼3 y. In addition, advocacy at the national and provincial levels targeted the extension of paid maternity leave to 6 mo, the strengthening of the Code of Marketing of Breast Milk Substitutes (including during the CF period), the reduction in stunting, and the improvement in provincial planning for IYCF and nutrition actions.

#### Evaluation design.

A cluster-randomized, nonblinded impact-evaluation design was used to compare the impact of the intensive and nonintensive A&T intervention packages. Cross-sectional household surveys were conducted at baseline (2010) and exactly 4 y later (2014) in the same communities among households with children aged 0–59.9 mo. This article presents findings on the core CF practices recommended by the WHO among children aged 6–23.9 mo and anthropometric outcomes in children aged 24–59.9 mo. The rationale for examining the impacts on growth among children aged ≥24 mo related to maximizing the time period of program exposure during the critical window of opportunity between conception and 24 mo of age. Although nutrition interventions should reach infants and young children during their first 2 y, the accrued impacts of these interventions should ideally be measured after the period of greatest potential benefit has concluded (after 2 y of age) (14).

#### Sample size estimations.

Sample size calculations were carried out to detect differences in the primary outcomes (i.e., CF practices among children aged 6–23.9 mo and stunting among children aged 24–59.9 mo) between the 2 intervention groups at endline, considering an α of 0.05, power of 0.80, an intraclass correlation of 0.01 (estimated from previous national or subnational surveys), and estimated baseline prevalence of the primary outcomes. We hypothesized that the intensive intervention would favor the primary impact indicators, and therefore a 1-sided test was used.

Assuming a baseline prevalence of 42% for minimum dietary diversity (25), we estimated that a total sample of 1000 infants aged 6–23.9 mo (500/group) was sufficient to detect a difference of ≥10 pps in the proportion of children achieving minimum dietary diversity at endline. In addition, a total sample of 2000 children aged 24–59.9 mo (1000/group) was sufficient to detect a difference of ≥6 pps in the proportion of stunted children at endline, assuming a baseline prevalence of 44%.

#### Randomization and blinding.

In Vietnam, 15 provinces (of 63) were selected for program implementation on the basis of stunting levels, absence of other large organizations working in nutrition, population density, and representation of the different ecological regions covered by the initiative. Four rural provinces, representing 4 distinct ecological zones, were then selected for inclusion in the evaluation sample. Ninety percent of franchises were at the commune level, and two-thirds were in rural areas. Given the large proportion of franchises in rural areas, and the homogeneity in sampling characteristics in rural areas, a decision was made jointly by the program and evaluation teams at the time the evaluation was being designed to focus the evaluation on rural franchises. Within these 4 provinces, 10 rural districts and 2–6 communes/district were selected for the evaluation on the basis of the presence of a health center that met the eligibility criteria for the A&T franchise model. Communes were randomly assigned to either the intensive (20 communes) or nonintensive (20 communes) intervention. The randomization process was carried out by using a simple public lottery system in the presence of local, district, and provincial health authorities as well as the program evaluators. Households within the intensive and nonintensive areas were not explicitly made aware of the results of the randomization. In addition, there was no blinding of the intervention at the level of service delivery.

#### Outcomes.

The primary outcomes were CF practices among children aged 6–23.9 mo, based on the indicators recommended by the WHO (26), and the prevalence of stunting among children aged 24–59.9 mo. Five CF indicators were examined: *1*) minimum dietary diversity (defined as children who consumed foods from ≥4 food groups out of 7 food groups in the previous 24 h), *2*) minimum meal frequency as appropriate for age, *3*) minimum acceptable diet (defined as children who were breastfed and who also achieved the minimum dietary diversity and age-appropriate minimum meal frequency), *4*) consumption of iron-rich or iron-fortified foods, and *5*) timely introduction of solid, semisolid, or soft foods (26). The total number of food groups consumed by the child in the previous day was also reported. Mothers or caregivers were asked about all liquids, solids, and semisolid foods consumed by the child during the previous day. Foods were then classified into different food groups on the basis of a standardized method for assessing IYCF practices (26). Anthropometric data were collected by using a standardized method (27). Weights of the children were measured by using electronic weighing scales that were precise to 100 g. Locally manufactured collapsible length and height boards, which were precise to 1 mm, were used to measure the standing height of children aged ≥24 mo. Weight and length or height were converted into height-for-age *z* scores, weight-for-age *z* scores, and weight-for-height *z* scores according to 2006 WHO child growth standards (28). Stunting, underweight, and wasting were defined as <−2 *z* scores for height-for-age, weight-for-age, and weight-for-height, respectively.

#### Statistical analysis.

Baseline differences between the 2 intervention packages were tested by using linear regression models (for continuous variables) or logit regression models (for categorical variables), accounting for geographic clustering (29). For impact analysis, we derived difference-in-difference impact estimates (DDEs) by using fixed-effects regression models that assessed differences between the 2 study groups over time (30) with the use of both intent-to-treat (ITT) analysis and a modified per-protocol analysis (MPA). The MPA was restricted to children whose mothers reported having visited an MTBT facility ≥1 time. There were not enough children who adhered to the recommended number of visits to an MTBT facility, as specified in the IPC package for CF, to conduct a usual per-protocol impact analysis among these children. Within the intensive group, we assessed differences in maternal and household-level characteristics among those who used MTBT services at least once and those who did not to investigate selection bias toward visiting an MTBT facility, either through their own agency or because of availability. We present DDEs, with adjustment for geographic clustering and infant age and sex, and also a fully adjusted model controlling further for those baseline characteristics that were different between groups and for those that changed differentially over time. To confirm the accuracy of self-reported outcome measures, we measured social desirability to assess potential biases in our main impact estimates on maternal reported CF practices by using the method described in the text accompanying **Supplemental Table 1**. Data analysis was performed by using Stata 13 (StataCorp). A statistical analysis plan was developed before endline data collection and discussed with the funder and program implementers.

#### Ethical approval.

Approval for the study was obtained from the institutional review board at the International Food Policy Research Institute and the Vietnam Union of Science and Technology Association. All mothers of the study children were provided with detailed information about the study in writing and verbally at recruitment. Written informed consent was obtained from all mothers. The study was registered at clinicaltrials.gov as NCT01676623.

## Results

### Trial flow and intervention duration

**FIGURE 1:**
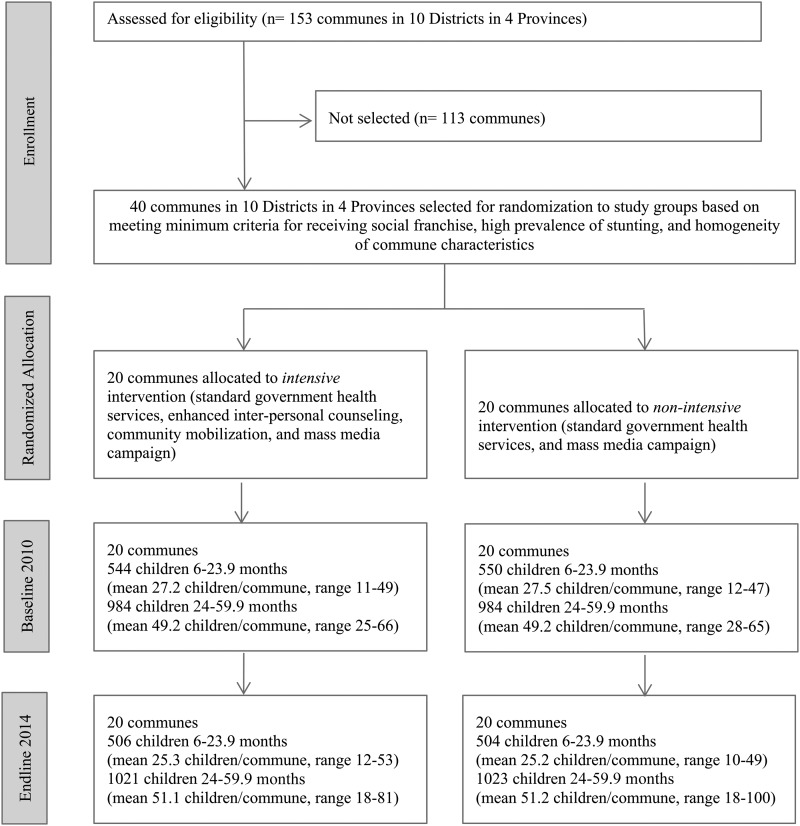
Trial profile.

No evaluation clusters were lost to follow-up (**Figure 1**), and none crossed from nonintensive to intensive during implementation. The cluster size varied across clusters and over time, reflecting commune population size.

### Sample characteristics

Because children in the 6- to 23.9- and 24- to 47.9-mo age groups were sampled separately, we present baseline characteristics of these 2 groups separately (**Table 2**). The randomization exercise was successful and resulted in a well-balanced set of key characteristics that might be related either to intervention uptake or intervention effectiveness. There were significant differences at baseline only for 2 household characteristics (ownership of agricultural land and ownership of garden) and number of prenatal visits. We accounted for these differences at baseline in our fully adjusted impact-estimate models.

**TABLE 2:** Selected characteristics of the study sample at baseline^1^

	Age 6–23.9 mo			Age 24–59.9 mo		
Characteristics	Intensive (*n* = 544)	Nonintensive (*n* = 550)	*P*^2^	Intensive (*n* = 984)	Nonintensive (*n* = 984)	*P*^3^
Household						
Children aged <5 y, *n*	1.2 ± 0.4	1.2 ± 0.4	0.22	1.2 ± 0.4	1.2 ± 0.4	0.28
Ownership of house, %	47.4	53.1	0.06	63.0	63.1	0.96
Ownership of garden, %	63.5	57.6	0.046	59.6	60.0	0.85
Ownership of agricultural land, %	71.1	73.5	0.38	72.2	76.4	0.03
SES^4^ index, *n*	−0.2 ± 1.0	−0.2 ± 0.9	0.95	−0.4 ± 0.9	−0.3 ± 0.9	0.06
Food security score^5^ (range: 0–27), *n*	2.2 ± 4.0	2.3 ± 4.0	0.70	2.7 ± 4.2	2.5 ± 4.2	0.55
Food insecurity,^5^ %	36.4	39.1	0.36	43.0	40.5	0.25
Household food dietary diversity, *n*	9.9 ± 2.3	9.9 ± 2.2	0.59	9.6 ± 2.5	9.9 ± 2.4	0.04
Household hygiene score (range: 0–10), *n*	7.2 ± 2.3	7.3 ± 2.2	0.52	6.6 ± 2.5	6.7 ± 2.4	0.39
Maternal factors						
Maternal stress,^6^ %	29.8	32.0	0.43	34.9	34.2	0.74
BMI, kg/m^2^	19.9 ± 2.5	19.9 ± 2.4	0.91	20.1 ± 2.5	20.1 ± 2.7	0.82
Education (range: 1–16 y), y	9.3 ± 3.4	9.4 ± 3.6	0.58	8.5 ± 3.4	8.8 ± 3.4	0.07
Occupation as farmer, %	53.9	52.4	0.62	61.2	56.7	0.04
Maternal dietary diversity, *n*	8.9 ± 2.3	9.0 ± 2.3	0.35	8.7 ± 2.5	8.8 ± 2.4	0.16
Health services access						
Prenatal visits, *n*	2.7 ± 2.7	2.4 ± 2.5	0.07	2.4 ± 2.6	2.0 ± 2.4	0.005
Mothers used any iron supplement during pregnancy, %	93.6	92.6	0.53	88.6	88.0	0.71
Child factors						
Female, %	46.9	46.7	0.96	49.3	46.3	0.18
Birth weight, kg	3.1 ± 0.5	3.1 ± 0.4	0.37	3.1 ± 0.5	3.1 ± 0.5	0.72
Age, mo	13.9 ± 5.3	14.4 ± 5.4	0.11	40.8 ± 9.7	40.7 ± 9.7	0.81
Acute respiratory infection,^7^ %	50.0	46.4	0.23	40.6	41.3	0.75
Diarrhea,^7^ %	13.3	13.7	0.85	6.0	7.1	0.33
Child anthropometric indicators						
HAZ	−0.8 ± 1.3	−0.8 ± 1.2	0.66	−1.4 ± 1.0	−1.3 ± 1.0	0.09
Stunting	15.3	14.3	0.62	25.4	22.5	0.14
WAZ	−0.8 ± 1.2	−0.7 ± 1.1	0.77	−1.2 ± 1.0	−1.1 ± 1.0	0.01
Underweight	12.2	9.9	0.22	20.5	16.2	0.01
WHZ	−0.5 ± 1.1	−0.5 ± 1.1	0.88	−0.6 ± 0.9	−0.6 ± 1.0	0.04
Wasting	5.4	5.9	0.72	5.3	4.1	0.20

1 Values are means ± SDs unless otherwise indicated. HAZ, height-for-age *z* score; SES, socioeconomic status; WAZ, weight-for-age *z* score; WHZ, weight-for-height *z* score.

2 *P* values for the difference between intensive and nonintensive at 6–23.9 mo.

3 *P* values for the difference between intensive and nonintensive at 24–59.9 mo.

4 An SES index was constructed by using principal components analysis with variables on ownership and assets. It is a standardized score with mean = 0 and SD = 1.

5 Household food security was measured by using the Food and Nutrition Technical Assistance III/US Agency for International Development’s Household Food Insecurity Access Scale.

6 Maternal stress was measured by using the WHO’s Self-Reporting Questionnaire–20 (31). We used a cutoff of 7 to classify women with high-level stress.

7 Acute respiratory infection and diarrhea were measured through maternal recall of symptoms in the 2 wk before the survey.

### Intervention exposure

The proportion of mothers with children aged 6–23.9 mo who reported ever being exposed to IPC at an MTBT health facility was 41.9%. In the 6 mo preceding the endline survey, reported exposure to IPC at an MTBT health facility was 39% (**Table 3**). In the intensive group, 23.1% of mothers reported having ever been exposed to IPC alone, 17.6% to MM alone, and 18.8% to both interventions. Among those who were exposed to IPC at an MTBT facility, the mean number of visits in the previous 6 mo was 1.3. Mothers who had visited an MTBT facility at least once (i.e., the modified per-protocol sample in the intensive group) had a greater intensity of exposure to MTBT services, overall and in the previous 6 mo, than did the full sample. Among women living in intensive areas who had visited an MTBT facility, the gap between actual and expected visits, on the basis of program design, increased with child age; this gap was widest among children aged ≥6 mo (results not shown**).** Although the average number of visits per month met the expected rate for pregnant women and women during delivery, the number of visits fell well below the expected number of visits among mothers of children aged 6–24 mo. Maternal-reported exposure to the television announcement promoting CF that was supported by A&T was 36% in intensive areas and 31% in nonintensive areas, with no significant differences between groups.

**TABLE 3:** Exposure to IPC and mass media among sample of mothers with children aged 6–23 mo at endline^1^

Indicator	Intensive (*n* = 506)	Nonintensive (*n* = 504)	Subset of intensive sample with any exposure to IPC (*n* = 212)
Ever exposed to IPC, %	41.9	—	—
Number of exposures to IPC, %			
0 visits	58.1	—	—
1 visits	17.6	—	42.0
2 visits	10.1	—	24.1
3 visits	5.9	—	14.2
≥4 visits	8.3	—	19.8
Average number of visits among those who were exposed to IPC	1.1 ± 1.8	—	2.5 ± 2.0
Exposure to IPC during the past 6 mo, %	39.3	—	73.1
0 visits	60.7	—	26.9
1 visits	18.6	—	34.0
2 visits	11.1	—	21.2
≥3 visits	9.7	—	17.9
Average number of visits during the past 6 mo	0.7 ± 1.0	—	1.3 ± 1.1
Exposure to CF TVC, %	36.4	30.6	44.8
Received invitation card (6–23.9 mo old), %	50.6	—	76.4
Exposure to IPC and mass media, %			
No exposure to either	40.5	69.4	—
Ever seen CF TVCs (only)	17.6	30.6	—
Ever exposed to IPC (only)	23.1	—	55.2
Exposed to both	18.8	—	44.8

1 Values are means ± SDs unless otherwise indicated. Only include exposures after 6 mo of age. CF, complementary feeding; IPC, interpersonal counseling; TVC, television commercial; —, data not available.

### Impact on CF practices

#### ITT analyses.

The measures of 4 core WHO CF indicators (minimum diet diversity, minimum meal frequency, minimum acceptable diet, and consumption of iron-rich foods) improved over time (*P* < 0.001 for all indicators) in both the nonintensive and intensive groups (**Table 4**). There was no differential improvement in favor of the intensive group; the DDEs of program impact on these 4 indicators were not significant in the pure ITT model or in the fully adjusted model.

**TABLE 4:** CF practices in the previous day among children aged 6–23.9 mo, by program and survey round in Vietnam^1^

	Baseline 2010 (T1)		Endline 2014 (T2)				
Impact indicators	Intensive	Nonintensive	Intensive	Nonintensive	DDEs^2^	Adjusted DDEs^3^	Fully adjusted DDEs^4^
ITT analysis							
Introduction of solid and semisolid foods	92.6	90.4	95.6	97.8	−4.4	−5.1	−4.9
Minimum diet diversity	73.7	75.8	90.9	87.9	5.1	4.61	4.0
Minimum meal frequency	79.8	82.0	95.5	93.3	4.5	4.4	4.4
Minimum acceptable diet	56.4	57.5	81.6	76.6	6.1	5.8	5.7
Consumption of iron-rich foods	85.8	85.3	97.2	95.6	1.1	0.5	0.6
Number of food groups (range: 0–7)	4.4 ± 1.6	4.4 ± 1.6	5.1 ± 1.2	5.0 ± 1.3	0.1	0.1	0.1
Number of meals (range: 0–13)	2.8 ± 1.0	2.8 ± 1.1	3.3 ± 1.1	3.3 ± 1.1	0.06	0.04	0.05
Modified per-protocol analysis^5^							
Introduction of solid and semisolid foods	92.6	90.4	92.7	97.8	−7.0	−8.0	−8.2
Minimum diet diversity	73.7	75.8	93.6^##^	87.9	8.0^+^	7.4*	6.4*
Minimum meal frequency	79.8	82.0	95.2	93.3	4.8	4.7	3.2
Minimum acceptable diet	56.4	57.5	86.9^###^	76.6	11.7*	11.5*	8.0*
Consumption of iron-rich foods	85.8	85.3	97.5	95.6	1.4	1.0	0.1
Number of food groups (range: 0–7)	4.4 ± 1.6	4.4 ± 1.6	5.3 ± 1.1^###^	5.0 ± 1.3	0.3^+^	0.3*	0.1
Number of meals (range: 0–13)	2.8 ± 1.0	2.8 ± 1.1	3.4 ± 1.1	3.3 ± 1.1	0.1	0.1	0.07

1 Values are means ± SDs or percentages unless otherwise indicated. ^+,^*Significant change from baseline to endline: ^+^*P* < 0.10, *P < 0.05. ^##,###^Different from nonintensive at that time: ^##^*P* < 0.01, ^###^*P* < 0.001. CF, complementary feeding; DDE, difference-in-difference estimate; ITT, intent-to-treat; MTBT, Mặt Tri Bé Th (The Little Sun); T, time.

2 DDEs with clustered SEs comparing intensive and nonintensive areas in 2010 and 2014, accounting for geographic clustering only. *P* values were obtained from regression models. DDEs for ITT analyses were for pure ITT analyses.

3 DDEs with clustered SEs comparing intensive and nonintensive areas in 2010 and 2014, accounting for geographic clustering, child sex, and child age. *P* values were obtained from regression models.

4 DDEs with clustered SEs comparing intensive and nonintensive areas in 2010 and 2014, accounting for geographic clustering, child sex, child age, and variables that were different at baseline (ownership of garden). Per-protocol analysis adjusted for ownership of garden, maternal education, and household food diversity. *P* values were obtained from regression models.

5 Modified per-protocol analysis included only women who had attended the MTBT at least once in the intensive group.

**FIGURE 2:**
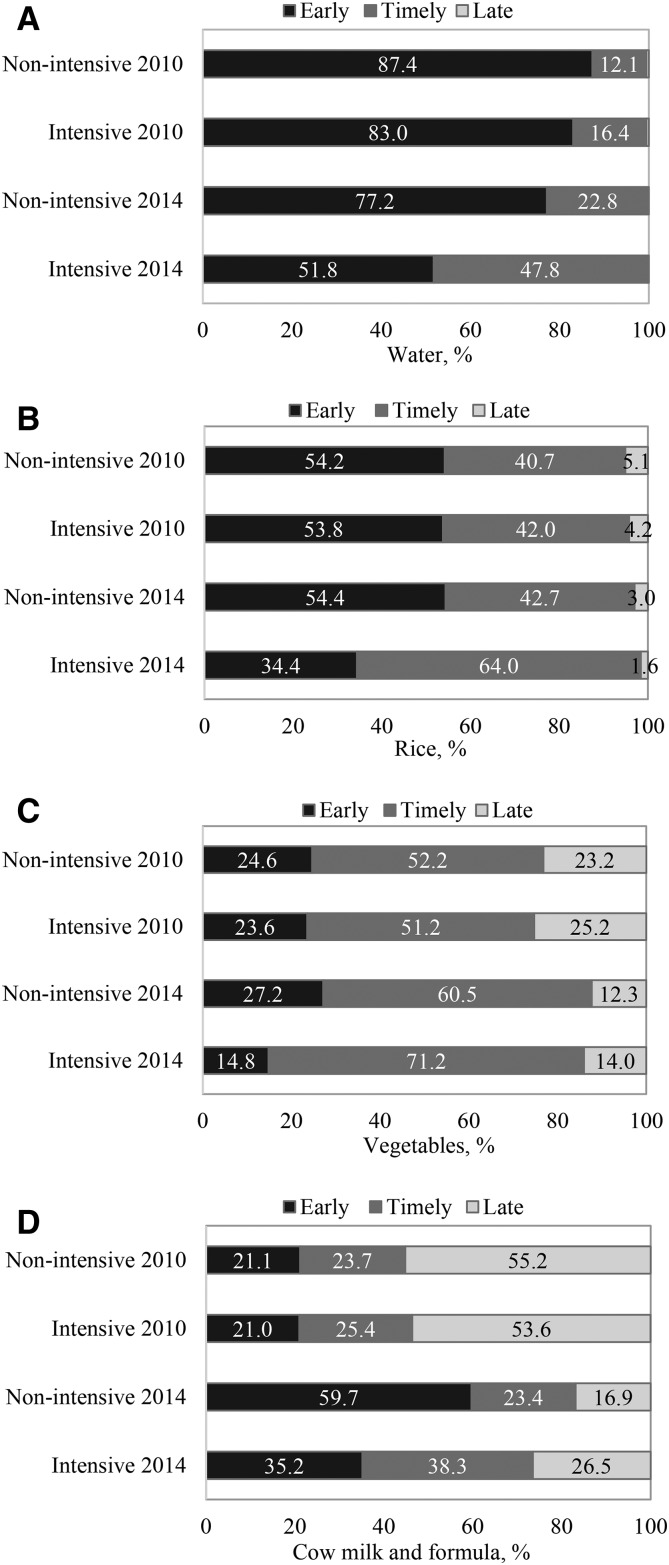
Timely food introduction, by program and survey round in Vietnam, for water (A), rice (B), vegetables (C), and cow milk and formula (D). The timely introduction of foods was defined as food introduced at 6–8.9 mo of age. Difference-in-difference estimates were 20.6, 20.2, 13.1, and 11.6 percentage points for water, rice, vegetables, and cow milk and formula, respectively.

There was a significant differential shift between groups from early or late introduction to a more timely introduction of water and other foods between the ages of 6 and 8.9 mo of age (**Figure 2**). These program impacts were large and significant, ranging from 12 to 21 pps for different foods. The shift was primarily from early to timely introduction for water and rice and from late to timely introduction for vegetables and other milks.

#### MPAs.

We found significantly higher maternal education, maternal and household food diversity, and ownership of a garden among those who had used MTBT services at least once, compared with nonusers (**Supplemental Table 2**). These covariates were included in our modified per-protocol DDE impact models. In this analysis, all core WHO CF indicators improved over time in the intensive group, and the increases were significantly higher in the intensive than in the nonintensive group for minimum dietary diversity (DDE: 6.4; *P* < 0.05) and minimum acceptable diet (DDE: 8.0; *P* < 0.01) (Table 4).

#### Assessing social desirability bias.

We found no evidence of a social desirability bias for any of the CF practices. In both the intensive and nonintensive groups, as social desirability scores increased there was no commensurate increase in the reported practice of CF for any indicator (Supplemental Table 2).

### Impact on stunting and other anthropometric indicators

Stunting declines occurred in both groups between baseline and endline, ranging from 5.4 pps in nonintensive group to 7.1 pps in the intensive group. No differential decline between groups was seen for the prevalence of stunting in the main impact-evaluation age group (24–59.9 mo; **Table 5**). This was the case in the pure ITT model as well as in adjusted models for both ITT analyses and MPAs. A similar pattern was observed for the proportion of children classified as being underweight and wasted. These overall nondifferential patterns in anthropometric indicators were also seen for children aged 6–23.9 mo (results not shown).

**TABLE 5:** Anthropometric indicators for children aged 24–59.9 mo, by program and survey round in Vietnam^1^

	Baseline 2010 (T1)		Endline 2014 (T2)				
Impact indicators	Intensive	Nonintensive	Intensive	Nonintensive	DDEs^2^	Adjusted DDEs^3^	Fully adjusted DDEs^4^
ITT analysis							
HAZ	−1.4 ± 1.0	−1.3 ± 1.0	−1.1 ± 1.0	−1.1 ± 1.0	0.1	0.1	0.1
Stunting	25.4	22.5	18.2	17.1	−1.7	−1.8	−1.1
WAZ	−1.2 ± 1.0	−1.1 ± 1.0	−0.8 ± 1.1	−0.8 ± 1.2	0.1	0.1	0.1
Underweight	20.5	16.2	12.4	11.4	−3.2	−3.2	−2.6
WHZ	−0.6 ± 0.9	−0.6 ± 1.0	−0.3 ± 1.1	−0.2 ± 1.2	0.03	0.03	0.01
Wasting	5.3	4.1	2.7	4.2	−2.7*	−2.7*	−2.5
Modified per-protocol analysis^5^							
HAZ	−1.4 ± 1.0	−1.3 ± 1.0	−1.1 ± 0.9	−1.1 ± 1.0	0.1	0.1	0.02
Stunting	25.4	22.5	19.4	17.1	−1.0	−1.3	0.01
WAZ	−1.2 ± 1.0	−1.1 ± 1.0	−0.9 ± 1.0	−0.8 ± 1.2	0.1	0.04	−0.02
Underweight	20.5	16.2	13.9	11.4	−1.9	−1.5	−0.8
WHZ	−0.6 ± 0.9	−0.6 ± 1.0	−0.3 ± 1.2	−0.2 ± 1.2	0.0	−0.02	−0.1
Wasting	5.3	4.1	2.6	4.2	−2.9^+^	−2.7^+^	−2.6

1 Values are means ± SDs or percentages unless otherwise indicated. ^+,^*Significant change from baseline to endline: ^+^*P* < 0.10, *P < 0.05. DDE, difference-in-difference estimate; HAZ, height-for-age *z* score; ITT, intent-to-treat; MTBT, Mặt Tri Bé Th (The Little Sun); T, time; WAZ, weight-for-age *z* score; WHZ, height-for-age *z* score.

2 DDEs with clustered SEs comparing intensive and nonintensive areas in 2010 and 2014, accounting for geographic clustering only. *P* values were obtained from regression models. DDEs for ITT analyses were for pure ITT analyses.

3 DDEs with clustered SEs comparing intensive and nonintensive areas in 2010 and 2014, accounting for geographic clustering, child sex, and child age. *P* values were obtained from regression models.

4 DDEs with clustered SEs comparing intensive and nonintensive areas in 2010 and 2014, accounting for geographic clustering, child sex, child age, and variables that were different in improvement at baseline and endline (maternal stress and household food dietary diversity for households with children aged 24–59.9 mo). Per-protocol analysis also adjusted for ownership of gardens, maternal education, BMI, socioeconomic status, and food security. *P* values were obtained from regression models.

5 Modified per-protocol analysis included only women who had attended the MTBT at least once in the intensive group.

## Discussion

In Vietnam, an at-scale program providing enhanced IPC in social franchise facilities through the government health system, together with CM and nationwide MM, did not have a significant impact on most CF practices measured or on child growth in ITT analyses. Overall, CF practices and child growth improved in both intensive and nonintensive areas, but these improvements were not different between groups in ITT analyses, with the exception of a differential shift between groups from early or late introduction to a more timely introduction of water and other foods between the ages of 6 and 8.9 mo. In MPAs with an inclusion criterion of having had ≥1 exposure to the franchise interventions, significant differential impacts were observed on key CF practices, including minimum dietary diversity and minimum acceptable diet.

To our knowledge, this is the first at-scale program that incorporates elements of social franchising to improve IYCF practices. Social franchising has increasingly been used in the health sector worldwide, with the majority of experience coming from reproductive health franchises, which have been shown to improve the availability, quality, and utilization of health care services (32, 33). Information on social franchising to address other health issues, including nutrition, is sparse. We previously reported on evidence that showed that incorporating elements of social franchising into government health facilities significantly enhanced the quality of nutrition counseling services (18) and had large, significant impacts on breastfeeding practices (17). Social franchising elements were built into the existing health care system without altering fundamental structures of health service delivery, such as staffing composition and the availability of services and providers.

The scale of this program and its primary focus on social behavior change communication (SBCC) distinguish it appreciably from previous studies and evaluations (3, 8–11) that examined feeding behavior and/or growth in relatively controlled settings (efficacy trials) primarily among families or children with confirmed receipt of an education- or food-based intervention and with high adherence rates. This evaluation, together with the evaluation of the A&T intervention in Bangladesh (14), is the first to our knowledge to measure and show significant improvements in CF at the population level in programs that operated at-scale and that focused predominantly on SBCC. During the 4-y intervention period, nearly 5000 frontline workers and health providers were trained, and the IPC reached an estimated 340,000 mothers of children aged <2 y in 15 of 63 provinces. The MM intervention operated at a national level, reaching >11 million women aged 15–35 y.

Although we found no differential impact on CF practices except for timely introduction in ITT analyses, the MPA, which used a low threshold for inclusion of having ever been exposed to MTBT services, did show a differential impact. We conducted an MPA because less than half of our endline sample had any exposure to IPC at an MTBT facility and only 40% had exposure in the previous 6 mo. In our MPAs, we controlled for those factors that were significantly different in the full intensive sample and in the subsample who were exposed to MTBT services to account for potential selection bias in the use of and access to MTBT services. The limited impact in ITT analyses appears to be primarily driven by low exposure to the intervention and utilization of the services.

The low exposure to MTBT services may have been driven to some extent by both program design and implementation factors that, in turn, influenced overall uptake. For example, the intervention was designed to focus on pregnant women and children ≤24 mo of age, but our process evaluation showed a stronger implementation focus on breastfeeding (during pregnancy and the first 6 mo of the child’s life) than on CF practices from 6 to 24 mo of age (34). This stronger focus on breastfeeding was justified, given the low rates of exclusive breastfeeding at baseline and the relatively high rates of optimal CF practices, and may have influenced the low uptake and use of program services among children aged ≥6 mo. Visits to an MTBT facility varied significantly by child age. The gap between the recommended number of visits (as per the design of the program) and actual visits was widest among children aged ≥6 mo (34). An important consideration influencing utilization in the context of Vietnam is that mothers tend to re-enter the workforce when their children are between 4 and 6 mo of age (35). This may be a critical constraint to utilization of facility-based services for preventive health care services and may affect the MTBT utilization rate for CF advice among mothers of children aged 6–24 mo. Thus, designing and using effective targeted demand-generation activities for preventive services are of critical importance to ensure that more women receive counseling (34).

The interventions tested in this evaluation focused predominantly on SBCC with the use of multiple components to deliver CF education and support. These were provided in the context of a relatively food-secure environment with access to diverse foods. Only one-quarter of our evaluation sample was classified as food insecure; at endline, ∼90% of children consumed ≥4 different food groups the previous day, as well as animal-source foods, in both the intensive and nonintensive areas. No additional interventions were provided that could have addressed other causes of constrained linear growth (1). The evidence to date on the impact of SBCC interventions to improve child growth is mixed, with some studies showing moderate impacts on linear growth (8, 11, 36) and others showing little to no effect (3). In our study population, it is possible that the general improvements in socioeconomic status (**Supplemental Tables 3** and **4**) observed during the study period in Vietnam (37) led to significant improvements in anthropometric indicators over time in both groups and that the interventions provided were unable to accelerate these improvements any further in the intensive group.

Some limitations of our program evaluation are important to acknowledge. First, the evaluation included only facilities that met the minimum criteria (i.e., dedicated, standardized counseling room; trained staff; availability of job aids and client materials). Our results may not represent what would have been seen for facilities not meeting the minimum criteria. Second, in this large-scale programmatic setting, for practical considerations we used a cross-sectional evaluation design that sampled children in 3 different age groups (0–5.9 mo to examine impacts on breastfeeding practices, 6–23.9 mo to examine impacts on CF practices, and 24–59.9 mo to examine impacts on stunting after being exposed to program interventions from birth until 23.9 mo of age) rather than tracking individual children. This design precludes our ability to fully link individual child–level exposure to program interventions with growth outcomes for the same children. Third, the intervention was not blinded to implementers or community members, which could have led to social desirability in reporting, especially since data on CF practices relied on maternal recall. We found, however, no intervention-specific differences in socially desirable reporting for CF practices (38). Fourth, we did not have the ability to assess the impact of IPC alone (compared with no intervention) because the media campaign was implemented nationwide. For these reasons, the impact estimates may underestimate the full potential of such a multipronged intervention because the evaluation lacks a “pure control” area. Last, because there was no group who received no intervention, we cannot determine whether the nonintensive intervention contributed to an impact on CF behaviors or whether this was a result of a secular trend.

There are several lessons learned from this novel social franchise approach to deliver high-quality IPC at scale and to improve CF practices, all within a government health system. The franchise start-up involved implementation steps to advance the model in 15 provinces simultaneously, ensuring that lessons learned could be broadly applied to other geographic areas. Extensive formative research was used to develop the behavior change interventions, including the IPC service package, the franchise brand, educational materials, and MM. The roll-out and scale-up of the franchise operations were carried out in conjunction with routine monitoring of quality and coverage, supervisory, and management approaches that supported implementation (39, 40).

In conclusion, this study provides rigorous evidence that shows that an innovative social franchising model that is integrated into the government health system in Vietnam and delivered at scale, along with an MM campaign and CM, improved CF practices when mothers were exposed to the intervention. Given that improvements were observed primarily among the subset of mothers who were exposed to the franchise at least once, it is important to address challenges to improve service utilization in order to achieve full program impact. For improvements in linear growth, it is likely that additional investments will be needed to accelerate current national trends in a food-secure context undergoing rapid socioeconomic and overall health improvements.

## Footnotes

8 Abbreviations used: A&T, Alive & Thrive; CF, complementary feeding; CM, community mobilization; DDE, difference-in-difference impact estimate; IPC, interpersonal counseling; ITT, intent to treat; IYCF, infant and young child feeding; MM, mass media; MPA, modified per-protocol analysis; MTBT, Mặt Trời Bé Thơ (The Little Sun); pp, percentage point; SBCC, social behavior change communication.
